# Testing citizen science as a tool for monitoring surface water microplastics

**DOI:** 10.1007/s10661-022-10487-w

**Published:** 2022-10-06

**Authors:** Outi Setälä, Jyri Tirroniemi, Maiju Lehtiniemi

**Affiliations:** Marine Research Centre, Finnish Environment Institute, Latokartanonkaari 11, 00790 Helsinki, Finland

**Keywords:** Manta trawl, Volunteer monitoring, Surface water, Spatial coverage, Sailing

## Abstract

The use of citizen science in the collection of surface water marine microplastics (MP) samples with manta trawl was tested in the Baltic Sea, where the collection of surface water samples is often hampered by environmental conditions. Sampling was carried out at 7 locations around the Baltic Sea with a custom-made manta trawl which was operated onboard a sailing boat. The total concentrations of ≥ 0.3 mm MP in the samples ranged from 0.45 to 1.98 MP m^−3^. Based on the results and experiences from this study, citizen science could be introduced into the toolbox of monitoring large MP. When the common basic constraints of surface water sampling within a regional sea are defined and agreed upon, citizen science could be used for strengthening the power of assessments on the state of the marine environment by increasing the spatial coverage of the monitored area.

**Supplementary Information:**

The online version contains supplementary material available at 10.1007/s10661-022-10487-w.

## Introduction

With the widespread increasing use of plastic products, the amount of plastic waste entering marine environments will continue to grow (Borelle et al., 2020). Plastics are long-lived and, once in the sea, can only partially be removed, if at all. The smallest plastic items, micro- and nanoplastics, will remain in the marine environment for an unknown time, which has caused concern of their potential ecological impacts. Microplastics are diverse small (< 5 mm; Arthur et al., 2009) plastic particles of varying shape, size, color, morphology, polymer composition which are coming from various anthropogenic sources. Microplastics have been found literally in all possible habitats, not only close to urban areas but also in places away from urban exposure, like in the deep sea (Woodall et al., 2014) or freshly fallen snow in Antarctica (Aves et al., 2021). Microplastics are either initially produced in their small size (Boucher & Friot, 2017) or being generated from larger plastic items during their use or weathering in the environment (Andrady, 2011; Beckwith & Fuentes, 2018; Song et al., 2017).

The exposure of biota is most likely through ingestion, which has been documented in animals throughout the marine food web, including zooplankton (Desforges et al., 2014), fish (Lusher et al., 2013; Sainio et al., 2021), marine mammals (Bravo Rebolledo et al., 2013; Lusher et al., 2015), and seabirds (Tourinho et al., 2010).

The ubiquitous distribution of MP together with their longevity in the environment, potential to slowly fragment into smaller and smaller pieces, and bioavailability to various marine organisms has during a relatively short time motivated multidisciplinary research and raised concern and interest worldwide, among researchers, policy makers, and citizens (SAPEA, 2019). The scientific community is still seeking a common understanding on how to carry out sampling, sample processing, and analyses of MP (Setälä et al., 2019), and how to categorize the particles (Hartmann et al., 2019; ECHA, 2019) by their material and size. The upper size limit is usually set to 5 mm or 1 mm (e.g., Hartmann et al., 2019), while the lower size limit varies more and depends on the research aims and the applied identification methods.

Microplastics are still a relatively new study object, and methods for estimating their presence in different environmental compartments have been developing for over a decade (e.g., Hanke et al., 2013). In order to plan and carry out successful management of marine litter and microplastics, data is needed on their amount, distribution, and sources. The cornerstone of marine litter monitoring in the European regional seas is the beach macrolitter surveys, which are carried out by the member states on designated beaches 3–4 times per year (Addamo et al., 2017). In many of the countries, these surveys have been initiated by non-governmental organizations (NGOs) who often also organize the local beach cleanups and surveys. For example, in the central Baltic Sea the first efforts to produce comparable data on beach macrolitter were made by NGOs from four Baltic Sea countries (MARLIN, 2013). The MARLIN project modified a beach survey protocol (Cheshire et al., 2009) for Central and Northern Baltic Sea environmental conditions, and this method with some modifications is still being used in 4 of the 9 Baltic countries. Recently, the task of streamlining common methodologies and categories for beach litter was carried out in collaboration with experts from different European countries (Addamo et al., 2018). Citizen science is suitable for the monitoring of beached macrolitter, if the quality of the data can be ensured (van der Velde, 2017). However, when it comes to the smallest size fractions of marine litter, i.e., microlitter and MP, citizen scientists are seldom involved (Hidalgo-Ruz & Thiel, 2015).

Like for beach litter, the need to monitor the distribution, amounts, and type of microlitter and MP stems from the EU Marine Strategy Framework Directive (MSFD) and its requirements (EC, 2008, 2017a, b). For the purposes of MSFD, plastic polymers should be identified from other anthropogenic materials. Microlitter consists of various materials, including artificial polymer particles, metal, glass, and paper. Although monitoring should cover all anthropogenic microlitter materials, focus has been on the development of sampling and sample processing methods for the plastic polymers.

Both sampling and sample processing of marine microplastics must be carried out with great care (e.g., Setälä et al., 2019). MP are found “everywhere,” and samples are easily contaminated with airborne particles and fibers. Contamination both during sampling and sample processing must be minimized and monitored with control samples. Also, all the equipment used in field and laboratory should be free from plastics, whenever possible. Rinsing the net well after sampling and storing it away from potential contamination sources, using organic clothes while sampling, and taking the wind direction into account when handling the net are all precautionary ways to avoid contamination. Instructions for interested people to carry out MP surveys http://www.seamarproject.org/images/projectos/MICROPLASTICS.pdf or building low-cost surface trawls are nowadays also found online: https://civiclaboratory.nl/citizen-science/.

When water MP are collected with a surface trawl, it is essential to measure the filtered water volume correctly. In calm sea conditions, the traveled distance may potentially be used to calculate the filtered water volume, if it is not possible to use a flow meter.

Methods used for assessing the distribution, amount, and type of MP are the most novel and include time-consuming working phases (e.g., Lastovina & Budnyk, 2021; Löder et al., 2017; Setälä et al., 2019). Especially the laboratory work, sample treatments, and material characterization may become a bottleneck, if the focus is towards the identification of smaller size fractions and detailed material characterization (Uurasjärvi et al., 2020). The processing of environmental MP samples is labor intensive and thus costly. The total costs for carrying out MP monitoring are formed from ship time, work onboard a vessel and in the laboratory, consumables, and in many cases new equipment which need to be purchased. The costs and time allocated for laboratory processes vary depending on the characteristics of the matrix studied (Mack et al., 2020).

Also, other issues than the direct costs of monitoring may limit the field sampling of MPs. In the study area of the present study, the Northern Baltic Sea, climate and environmental biotic conditions set frames for the suitable sampling season. Presently, the most common method for collecting data on MPs in water is to use a surface manta trawl, although also different methods, such as underwater pumps (Karlsson et al., 2019; Setälä et al., 2016), bulk sampling (Uurasjärvi et al., 2019), multinet-type and vertical net sampling (Reissser et al., 2015; Uurasjärvi et al., 2020) have been applied. Using a trawl may not always be the best option, or even be possible. Wind and wave height together with the presence of organic material in the water are the main parameters limiting MP sampling from water. In the Baltic Sea the mean wind speeds are commonly high in autumn and during early winter, while late spring and early summer are usually the seasons for calm weather (Jönsson et al., 2002). Summer would thus be the ideal season for towing a surface trawl. However, not only the phytoplankton spring bloom, but also vigorous algal blooms in autumn limit manta trawl sampling. Wintertime is left out, not only because of higher probability for strong winds, but also because of the seasonal ice cover during winter at least for some parts of the sampling area. These limitations play a role, when manta trawl sampling in the northern parts of the Baltic Sea is planned.

Data should be collected to evaluate the overall distribution of MPs, and as advised, preferably also to pinpoint potential sources of microlitter pollution. Because most of the sources are due to land-based human activities, and the small light-weighted litter particles are rapidly dispersed in the water, both open sea areas and sites closer to the probable land-based sources of MP and their pathways (Miller et al., 2021) should be included in the sampling scheme. If the overall costs can be lowered, the spatial coverage could potentially be enlarged. Citizen science offers solutions for collecting data for larger scale assessments (Rambonnet et al., 2019) and expanding spatial coverage (Forrest et al., 2019). Designated manta trawls have already for some years been used by various non-profit organizations and other stakeholders to collect data on the distribution of MP from the sea surface (e.g., UNEP, 5Gyres, Foundation Taraocean, by the Oceans we Unite, Greenpeace).

In the Baltic Sea, the use of citizen science for collecting surface water MP was first presented in a study by Gewert et al. (2018), which included not only data collected by trained researchers, but also citizen science data collected by two adventurers who traveled with standup paddleboards towing small nets behind the boards. After analyzing the results, the authors concluded that the quality of data was relatively good. There had been no special problems regarding contamination, which is always the main concern, but as a drawback the actual filtered water volume was not possible to measure. However, the general conclusion was that citizen science might potentially be used for collecting data on MP to support the knowledge base on MP distribution. One possibility to increase the amount of collected data is thus to engage citizens not only to macrolitter but also MP monitoring. With careful planning, field samples could be collected with the help of active citizens and consecutively process the samples in research laboratories the same way as “official” monitoring samples.

This pilot study was initiated as a collaboration with the researchers from the Marine Research Centre of the Finnish Environment Institute (SYKE) responsible for the national monitoring program of MP and a Finnish sailor-adventurer (K. Nurmi). The aim of this study was to test if MP sampling by citizens carried out on leisure boats could potentially be used as a tool for surface water MP monitoring to complement national monitoring carried out by researchers.

## Material and methods

### Sampling and equipment

**Fig. 1:**
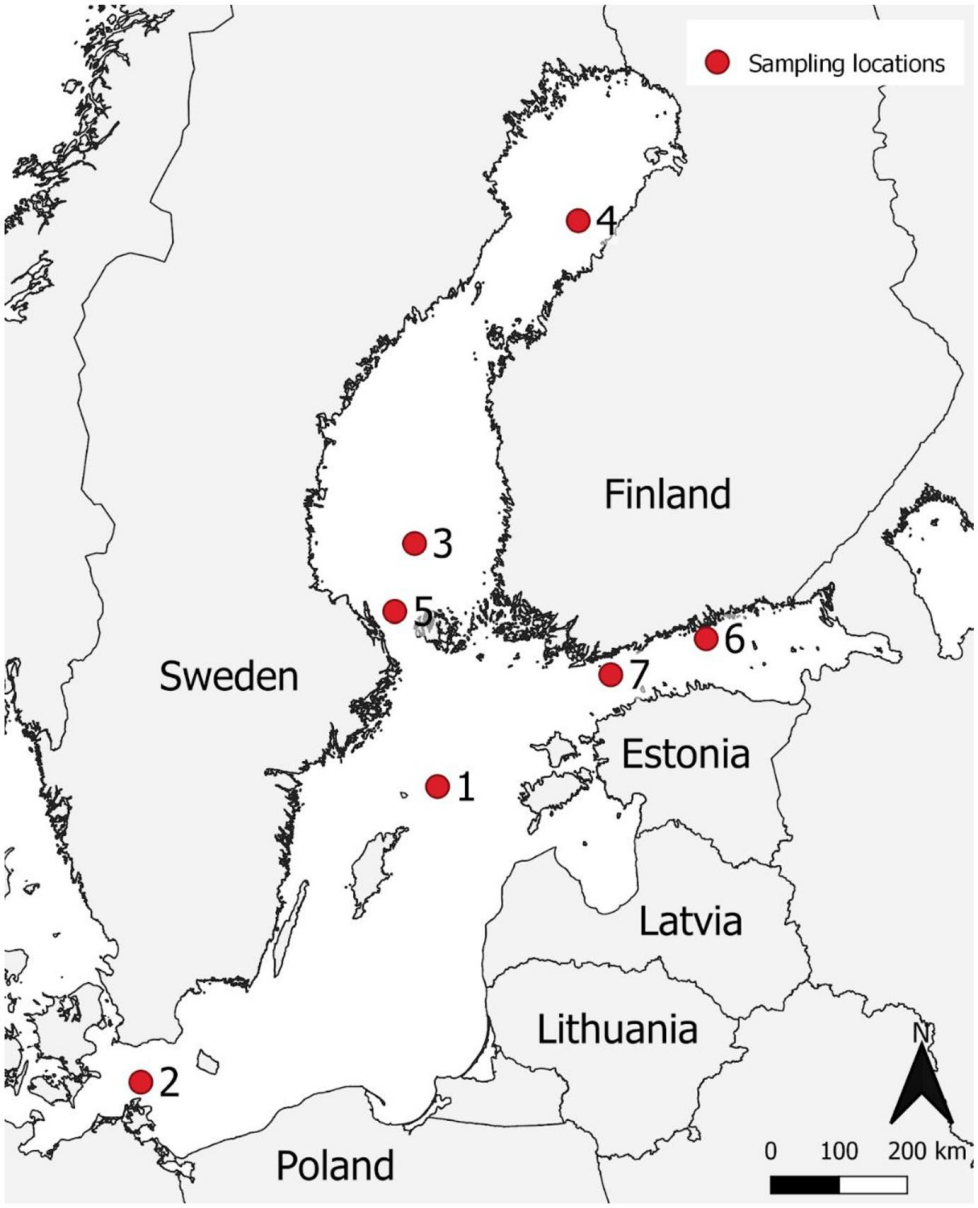
Sampling locations in the Baltic Sea by the sea basins: 1. Northern Baltic proper, 2. Arkona Basin, 3. Bothnian Sea, 4. Bothnian Bay 5. Åland Sea, 6. and 7. Gulf of Finland

**Fig. 2:**
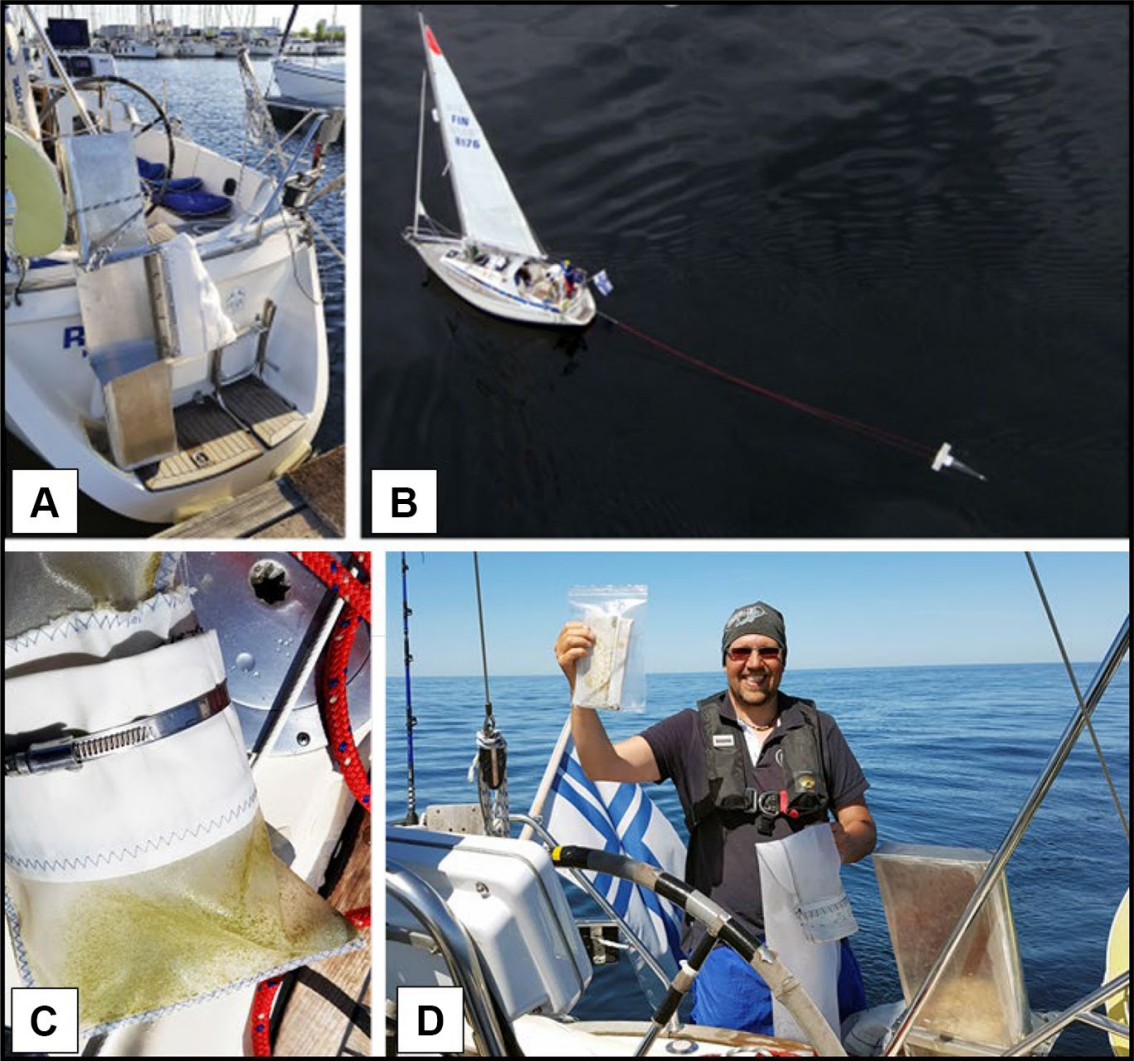
Sampling: **A**) manta trawl attached to the sailing boat, **B**) towing the mini-manta trawl, **C**) sampled material in the cod end, **D**) stored sample in a ziplock bag. Photo credits: **A** and **C**: Kari Nurmi, **B** and **D**: Esa Kekki

Samples were collected in May–June 2018 from 7 locations around the Baltic Sea (Fig. 1). Sampling was carried out with a 9-m-long sailing boat which sailed non-stop altogether 2700 nautical miles in the Baltic Sea. Because free space for storing equipment was limited, a small version of the manta trawl was constructed (Fig. 2A). The dimensions of this “mini-manta” were: 460 mm × 133 mm (mouth), 445 mm × 273 mm × 54 mm (wing), and the mesh size of the net was 0.3 mm (see the supplementary material for details).

Sampling was carried out with an average travel speed of 1.5 knots by towing manta trawl for 30 min. An outboard motor was used during towing to keep the travel speed constant (Fig. 2B). The location of the boat at the start and end of sampling was registered with a GPS, and the distance sailed was used to estimate the filtered water volume. Each collected sample was collected into a pre-cleaned cod end (Fig. 2C), which was removed after trawling and placed immediately in a ziplock bag (Fig. 2D) and preserved with commercially available 40% ethanol. The reasons for using ethanol were simply the lack of freezer/cold storage onboard and the easy accessibility of ethanol for the potential future citizen scientists. Because no water pump for washing the trawl was available, the trawl was rinsed simply by towing it without the cod end after every sampling. A new pre-cleaned cod end was attached to the trawl right before the next sampling. After the cruise, the ziplock bags containing the samples were brought to the laboratory and stored at + 5 °C until processed further. No field control samples were taken.

### Sample treatment and analyses

The samples were processed in the laboratory facilities of the Finnish Environment Institute which is specialized in processing of environmental MP samples. To prevent sample contamination, all laboratory equipment and materials were thoroughly rinsed with distilled water before use, and the chemicals were filtered through a 0.7-μm GF/F filter before use. Cotton lab coats were worn throughout the work, and all work steps were done in a laminar hood. Each sample was carefully washed out from the respective cod end with Milli-Q water into a transparent plastic jar, covered with aluminum foil, and frozen in − 20 °C prior to the next step of sample processing: digestion of organic material. Prior to the digestion, the water samples were defrosted in room temperature. The extraction of MP from the samples followed the stepwise enzymatic digestion protocol of Löder et al. (2017). The chemicals used were (in this order): sodium dodecyl sulfate (SDS), protease, cellulose, hydrogen peroxide, chitinase, hydrogen peroxide, and zinc chloride. Whole sample volume was filtered into one 0.1-mm nylon filter. Blank samples were used to follow airborne contamination. No contamination of particles ≥ 0.3 mm was found from the laboratory controls.

MPs were identified and counted using Leica M165 stereomicroscope with white light on. Stereomicroscope was applied for the analyses of these samples because the large size of the MPs (≥ 0.3 mm) allows identification with optical microscopy (Setälä et al., 2019). Because the samples were collected after the spring bloom and further treated with a digestion protocol, only some remains of organic material were left which could have disturbed the analyses. All the particles were photographed, counted, and measured along the longest diameter using Leica application suite software. The particles were confirmed as plastics with a hot needle test where a heated needle is pushed against the particle’s surface and changes in particle morphology are observed. Fibers and particles were categorized into six size classes (0.3–1, > 1–2, > 2–3, > 3–4, > 4–5 mm, and > 5 mm). Confirmed plastics were further divided into particles and fibers visually.

## Results and discussion

**Fig. 3:**
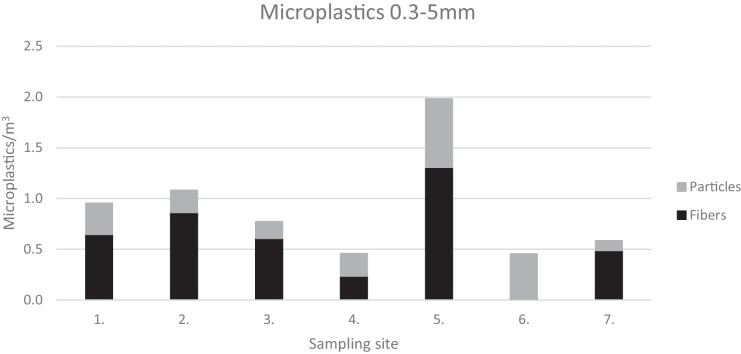
MP concentration by sampling location

**Fig. 4:**
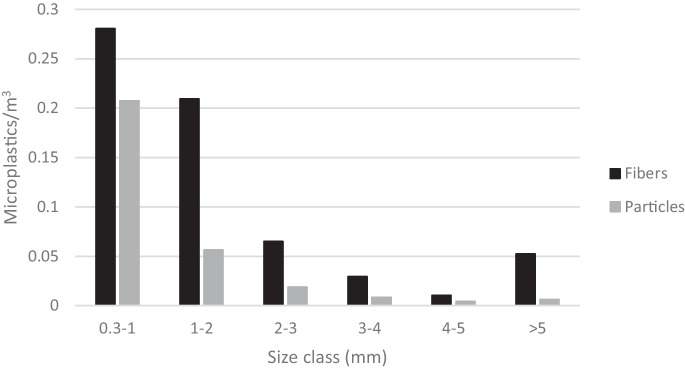
The concentration of MP types (particles and fibers) in different size fractions (all samples pooled together)

The distance traveled during one trawl tow varied between 1227 and 1684 m, and the estimated filtered water volume varied accordingly, from 59.7 to 81.8m^3^. The total MP concentration (≥ 300–5000 µm) in the samples varied between 0.45 and 1.98 MP m^−3^ (Fig. 3). Highest concentrations were found from the sampling site 5 in the Åland Sea, an area with busy maritime traffic. In overall, the concentration of MP increased towards the smallest size fractions (Fig. 4).

**Fig. 5:**
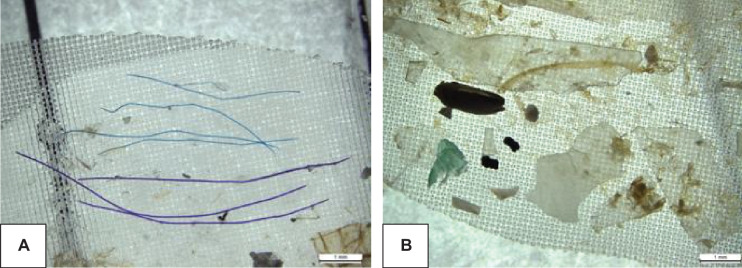
Fibers **A**) and different MP particles retrieved from the samples

The sample material contained particles larger than 5 mm, which is commonly used as the higher size limit for microplastics, as well as particles that were smaller than the mesh size of the manta trawl. Plastic fibers were mostly dark (Fig. 5A), while the particles (Fig. 5B) varied in texture and size, as well as color, majority of them being, however, white, opaque, or clear. Most of the MP were > 0.3 mm, but occasionally also smaller items were found, usually attached to other material.

Fibers outnumbered particulate MP in the above-mentioned size groups (51.7–83.3%), at all other sampling sites except in the eastern Gulf of Finland (site 6), where the proportion of fibers was negligible (3.4% of all MP) (Fig. 3). However, while the concentration of total MP decreased with increasing size fraction (Fig. 4), the proportion of fibers remained higher than the proportion of particulate MP and was actually highest in the largest size fraction which is outside the commonly used definition (e.g., Hartmann et al., 2019) of MP (> 0.3–1 mm: 57.5%; > 1–2 mm: 78.7%, > 2–3: 77.5%, > 3–4 mm: 77.8%, > 4–5 mm: 71.4%, > 5 mm: 89.3%). The material of the towing rope was synthetic, and it is possible that some of the fibers in the samples were originating from the rope. In any case, the results regarding fibers do not reflect the actual fiber concentrations in the water, when a manta trawl is applied, since the mesh size of the trawl is so big that it allows some of the fibers to pass through. Some fibers, however, usually remain in the samples, often forming tight clumps. Although not quantitative, the information on fibers can in any case be used for assessing the sources of MP.

Data is piling up but do not yet allow a thorough assessment of the level of MP contamination in the area because of its heterogeneity, although the distribution, amount, and type of MP in water surface have been studied in the Baltic Sea area already over a decade (Table 1). These results of the present study do not differ markedly from the previous results obtained by professional researchers from the study area, where surface water MP concentrations between 0 and 0.8 m^−3^ have been detected. Higher concentrations have also been detected. The study of Gewert et al. (2018) from the Stockholm archipelago, east coast of Sweden, found higher MP concentrations close to highly populated coastal areas. Relatively high MP concentrations (16.2 ± 11.2 MP m^−3^) were also found in the study of Sainio et al. (2021) from the Finish coast, where surface water samples were collected with a pump instead of a trawl. A recent study of Zhou et al. (2021) from the Baltic Sea reports much higher MP concentrations at all depths sampled between deep water layers and water surface, and accumulation of MPs (up to 27,700 items m^−3^) in subsurface water layers. This dataset includes MPs in the size range of 27.3–5000 µm, most of them being identified as fibers (90.4%). Unlike most previous studies, Zhou et al. (2021) used a small volume water sampler for collecting the samples and did not apply digestion protocols. A water sampler (30L) was also used in the study of Uurasjärvi et al. (2020), where accumulation of MPs (ca 440 MP m^−3^) at haloclines was detected. Like Zhou et al., this study included MP in smaller particles (< 50 µm) than what can be collected with a common manta trawl, but as a difference, Uurasjärvi et al. (2020) applied a digestion protocol and found a low number of fibers in the samples (2.4%). However, no samples from water surface was taken for this study. Because the methods for marine MP monitoring have been under development, different studies have applied varying methods for sampling and/or sample processing. It is obvious that comparing the results from different studies may after all not be reasonable, since different sampling techniques provide different type of data. Also, the applied digestion protocols, or lack of them, have their effects on the results.

**Table 1:** Field collected data on surface water MP concentrations in the Baltic Sea

Study area	Sampling methods	MP/m^3^	Size (µm)	Analytical method	Reference
Swedish coast (Baltic Sea and North Sea)	Submersible pump	0–2.5	300–5000	Optical microscopy (stereomicroscope)	Magnusson and Norén (2011)
Gulf of Finland	Manta trawl	0.3–0.7	300–5000	Optical microscopy (stereomicroscope)	Magnusson (2014)
Gulf of Finland	Pump	0.10–6.5 (fibers) 0.5–9.4 (particles)	≥ 20, filtered with a sandwich type filtering tower: (20 and 100)	Optical microscopy (stereomicroscope)	Talvitie et al. (2015)
Gulf of Finland	Manta trawl Submersible pump Submersible pump	0–0.8 0–1.25 0–6.8	≥ 333 ≥ 300 ≥ 100	Optical microscopy (stereomicroscope)	Setälä et al. (2016)
Stockholm archipelago	Manta trawl	0.19–7.73	≥ 335	Optical microscopy (stereomicroscope), confirmation of selected particles spectroscopically (µFTIR)	Gewert et al. (2018)
South Funen archipelago, southern Baltic Sea/Kattegat	Manta trawl	0.05–0.09	≥ 300	Image analysis of Nile Red stained samples	Tamminga et al. (2018)
Western Gulf of Finland	Pump	0.7–1.3 0.3 22	≥ 300 100–300 20–100	Optical microscopy (stereomicroscope)	Railo et al. (2018)
Skagerrak/Kattegat, Gulf of Bothnia	Manta trawl Pump Pump	0–0.46 0–10.5 0–70.3	> 300 > 300 > 50	Optical microscopy (stereomicroscope) + near infrared hyperspectral imaging	Schönlau et al. (2020)
Baltic Sea (2 stations)	Manta trawl	0.019 and 0.022	> 335	Optical microscopy (stereomicroscope) + confirmation of selected particles spectroscopically (µFTIR)	Hänninen et al. (2021)
Finnish coast, northern Baltic sea	Pump	16.2 ± 11.2	> 100	Optical microscopy (stereomicroscope)	Sainio et al. (2021)
Baltic Sea	Rosette (approx. 10L), 5L subsamples	0–11,850	< 100–1000 (water sample filtered onto 5-µm pore size filter)	Optical microscopy (stereomicroscope) + confirmation of selected particles spectroscopically (µFTIR)	Zhou et al. (2021)
Northern Baltic Sea and Arkona basin	Mini-manta trawl	0.4–1.7 0–0.2	300–5000 ≥ 5000	Optical microscopy (stereomicroscope)	This study (citizen science)

The monitoring results are affected by sampling and sample processing, as well as the applied analytical methods. When water samples are being collected, the mesh size of the net, or filter, is the primary constraint affecting the results. When a surface trawl is used, the size and shape of the mouth opening can have an impact on the number and size of particles captured (Eriksen et al., 2018). Also, the towing speed is an important factor affecting both the filtered water volume through the traveled distance, but also as too high speed will cause the trawl pushing water in front of the mouth opening decreasing the sampling efficiency by pushing particles through the net or damaging them (Eriksen et al., 2018).

Secondly, the water volume which is filtered must be acceptable in order to capture a representative number of particles (Karlsson et al., 2019). This may be a problem in environments where the amount of organic material limits the towing time because of clogging. In such a case, the spatial coverage remains low, and the number of particles probably too low for any statistical analysis. On the other hand, in some occasions sampling during times of high organic material in the surface may increase the relative number of captured particles per filtered water volume compared to clear water seasons, if MP are attached to organic material such as algae. The samples may also contain MP which are entangled in the organic material, even though they are smaller than the actual mesh size. For overcoming such problems, optimal sampling conditions should be defined together with researchers, not only the quality assurance and control for the sampling and sample processing. For using a surface trawl, limits for the wind speed and/or wave height should be identified, as well as the minimum volume of water filtered and the justification for time intervals when the cod end of a trawl should be emptied, if longer transects are being monitored.

Bearing in mind the limitations when comparisons are made, it however seems that the results from the present study do not markedly differ from the results from previous studies from the Baltic Sea, which have been conducted by professional researchers (Table 1). The reason probably is that most of the particles identified have been relatively large (> 100–300 µm).

When such relatively large MPs are studied, citizen science could thus be a tool for increasing the spatial coverage of monitoring, by for example reaching shallow coastal sea areas where monitoring vessels cannot operate. Also, sampling by citizens is more flexible. Compared to monitoring cruises which usually have tight pre-arranged schedules, sampling by citizens can be arranged at times of most suitable sampling conditions. Sampling of MP does not require special skills, but the individuals conducting the work need to be aware of some important constraints, especially of how to minimize contamination during sampling. If citizens are to be engaged in the sampling of surface water MP, they need to be trained in advance, and a protocol for sampling should be designed together with researchers. In our study the sampling was planned together with the person in charge of the sailing boat before the start of the trip, and the manta trawl was custom-made based on the practical limits of the boat. We were aware that some of the prerequisites might not be 100% fulfilled, since not all the facilities and equipment commonly used during surface net sampling (a winch, running water, freezer) were available. Especially the lack of running water was identified as a potential problem, since it was not possible to rinse the trawl and the cod end after sampling. This was partially solved by providing several cod ends and storing the sample inside the cod end. This way also contamination from handling the net and cod end in the field was lowered. It was not possible to wash the net between the sampling tows. If plankton material is not rinsed properly from the net, the mesh gets clogged, and this affects the quality of the collected material. Also, some of the collected microplastics may get entangled in the organic material. This was solved by towing the net empty after each tow, which was possible only because there was not much organic material in the water at the time of the sampling.

The aim of this study was to test whether citizen science could be used in collecting samples for MP monitoring, and if these samples could be used to complement the national monitoring data. Based on this limited dataset we conclude that citizen science could potentially be used for collecting additional data, especially if relatively large MP or potentially even meso-sized plastics are being monitored. Similar conclusions have previously been made in studies where beach sand has been collected by citizens for microplastic analyses (Besley et al., 2017; Bosker et al., 2017). In the study of Bosker et al. (2017) the authors conclude that the collection of samples does not affect the outcome of the results, as the actual sample processing and analyses are carried out with standard methods by expert researchers.

When field sampling is being planned, the leading question would not be who is doing the field surveys, but rather which type of information is needed and how it can be gathered in the most efficient way. In overall, there the value of citizen science lies in the increased spatial coverage of samples, to better reveal the spatial differences in MP distribution and to gain information on potential sources and pathways of MP. However, the present study also showed that surface water sampling has more limitations than for example beach, or sediment sampling. Seasonal limitations in sampling need to be considered. Because the sampling was carried out in June at a time after the vigorous spring phytoplankton bloom had already ceased, it was possible to clean the trawl after sampling by simply towing it without the cod end. If there would have been a bloom, this maneuver would not have been adequate for clearing the net, which would likely have been clogged with phytoplankton. However, with careful planning together with citizen scientists and researchers, such problems can be avoided. Special care should be taken in defining accurately the filtered water volume (preferably using flow meters), washing the net after each sampling (access to a harbor or using a vessel with running water), applying all precautionary methods for ruling out contamination (using metal wire or rope made of organic material for towing), wearing clothes made of organic materials, taken into consideration the wind direction while maneuvering the trawl, storing samples in the cod ends, and preparing control samples also during the field work not only in the laboratory. Sampling should only be carried out in suitable environmental and weather conditions.

## Conclusions

Microplastics are included in the list of the parameters which must be monitored within the EU Marine Strategy Framework Directive. The present methods used for processing environmental samples and analyzing their MP numbers and types are time-consuming, expensive, and require special expertise. In contrast to that, field sampling in most cases is relatively straightforward, and successful sampling can be carried out independently after supervision by researchers. The study shows that well planned and supervised sampling by citizens can provide good quality samples of MP from surface waters, which can further be used to complement monitoring, especially in the northern Baltic Sea, where the time window for MP monitoring from surface waters is restricted due to intensive algal blooms during late summer and harsh weather conditions and ice cover during autumn and winter. In addition, citizen science also offers opportunities for lowering the overall costs of marine litter monitoring and increasing the spatial coverage of monitoring.

## Supplementary Information

Below is the link to the electronic supplementary material.

Supplementary file1 (DOCX 157 KB)

## Data Availability

The datasets generated during and/or analyzed during the current study are available from the corresponding author on reasonable request.
