# Supplementary material for: Testing citizen science as a tool for monitoring surface water microplastics
Source: Environ Monit Assess. 2022 Oct 6;194(12):851. doi: 10.1007/s10661-022-10487-w (PMC9537197; doi:10.1007/s10661-022-10487-w)
Supplement: Supplementary file 1 — Supplementary file1 (DOCX 157 KB) [file 10661_2022_10487_MOESM1_ESM.docx]

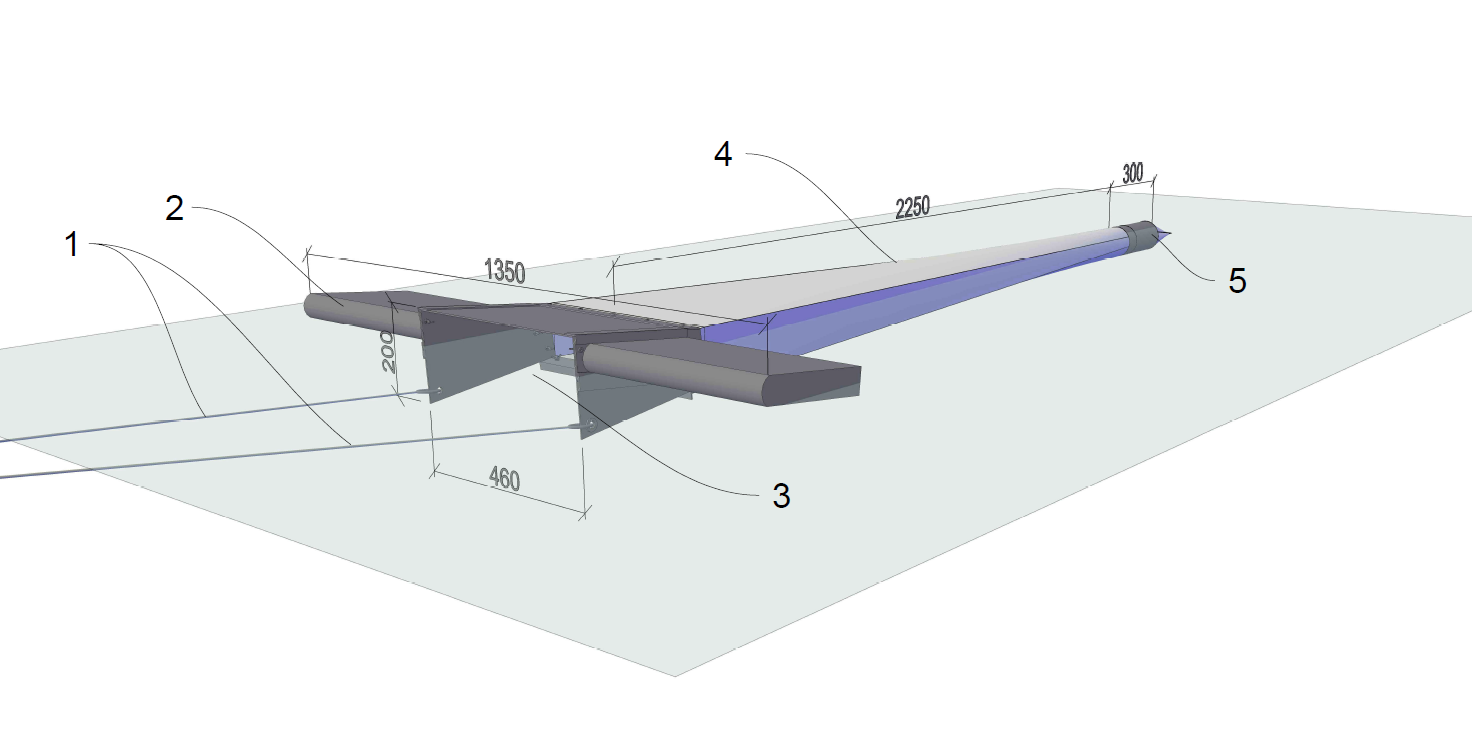


**Supplementary material**

Schematic drawing of the mini-manta trawl. Ropes for towing

1. Wings (hollow)
2. Mouth opening
3. Net
4. Cod end
